# Inhibition of store-operated calcium entry in microglia by helminth factors: implications for immune suppression in neurocysticercosis

**DOI:** 10.1186/s12974-014-0210-7

**Published:** 2014-12-24

**Authors:** Yuyang Sun, Arun Chauhan, Pramod Sukumaran, Jyotika Sharma, Brij B Singh, Bibhuti B Mishra

**Affiliations:** Department of Basic Sciences, School of Medicine & Health Sciences, The University of North Dakota, 501 N Columbia Road, Grand Forks, ND 58202 USA

**Keywords:** Calcium signaling, Helminth, Immune suppression, Microglia, Neurocysticercosis, Neuroinflammation, ORAI1, Store-operated calcium entry, TRPC1

## Abstract

**Background:**

Neurocysticercosis (NCC) is a disease of the central nervous system (CNS) caused by the cestode *Taenia solium*. The infection exhibits a long asymptomatic phase, typically lasting 3 to 5 years, before the onset of the symptomatic phase. The severity of the symptoms is thought to be associated with the intensity of the inflammatory response elicited by the degenerating parasite. In contrast, the asymptomatic phase shows an absence of brain inflammation, which is presumably due to immunosuppressive effects of the live parasites. However, the host factors and/or pathways involved in inhibiting inflammation remain largely unknown. Recently, using an animal model of NCC in which mice were intracranially inoculated with a related helminth parasite, *Mesocestoides corti*, we reported that Toll-like receptor (TLR)-associated signaling contributes to the development of the inflammatory response. As microglia shape the initial innate immune response in the CNS, we hypothesized that the negative regulation of a TLR-induced inflammatory pathway in microglia may be a novel helminth-associated immunosuppressive mechanism in NCC.

**Methods and results:**

Here we report that helminth soluble factors (HSFs) from *Mesocestoides corti* inhibited TLR ligation-induced production of inflammatory cytokines in primary microglia. This was correlated with an inhibition of TLR-initiated upregulation of both phosphorylation and acetylation of the nuclear factor κB (NF-κB) p65 subunit, as well as phosphorylation of JNK and ERK1/2. As Ca^2+^ influx due to store-operated Ca^2+^ entry (SOCE) has been implicated in induction of downstream signaling, we tested the inhibitory effect of HSFs on agonist-induced Ca^2+^ influx and specific Ca^2+^ channel activation. We discovered that HSFs abolished the lipopolysaccharide (LPS)- or thapsigargin (Tg)-induced increase in intracellular Ca^2+^ accumulation by blocking the ER store release and SOCE. Moreover, electrophysiological recordings demonstrated HSF-mediated inhibition of LPS- or Tg-induced SOCE currents through both TRPC1 and ORAI1 Ca^2+^ channels on plasma membrane. This was correlated with a decrease in the TRPC1-STIM1 and ORAI1-STIM1 clustering at the plasma membrane that is essential for sustained Ca^2+^ entry through these channels.

**Conclusion:**

Inhibition of TRPC1 and ORAI1 Ca^2+^ channel-mediated activation of NF-κB and MAPK pathways in microglia is likely a novel helminth-induced immunosuppressive mechanism that controls initiation of inflammatory response in the CNS.

## Introduction

Neurocysticercosis (NCC) is the most common parasitic infection of the central nervous system (CNS) caused by the cestode *Taenia solium*. It affects 50 million to 100 million people worldwide [1]. As many as 50% of adult-onset seizures and 10% of the stroke cases in endemic areas are attributed to NCC [2,3]. Treatment of NCC remains a major challenge, as the severity of the symptoms is thought to be due mainly to the inflammatory response elicited by the degenerating larvae resulting from therapeutic treatment or normal attrition [2,4]. Interestingly, a long asymptomatic phase (lasting for years) precedes the onset of the symptomatic phase. This asymptomatic phase is characterized by little to no sign of inflammation detected around the live cysts [2,5,6]. One possible explanation could be that viable cysticerci induce immunosuppressive effects to evade the host immune response. Furthermore, loss of these effects (when the parasite dies) could lead to uncontrolled hyperinflammatory responses that contribute to tissue pathology and clinical signs and symptoms such as severe headaches, epilepsy, intracranial hypertension, focal deficit and cognitive impairment [2,5,6]. However, the mechanisms underlying the immunosuppressive events remain poorly understood.

The regulation of inflammatory responses is mediated mainly via the innate immune system through myeloid antigen-presenting cells, such as macrophages and dendritic cells. In the CNS, microglia are the native myeloid cells that shape the initial innate immune response [7]. These cells recognize external stimuli through a wide variety of surface receptors, which culminates in release of proinflammatory cytokines and/or chemokines and effector molecules such as the antimicrobial molecules and reactive oxygen and reactive nitrogen intermediates [7–9]. Generally, the induced inflammatory response promotes the destruction of pathogens, but it can cause widespread tissue damage as a result of their overactivation [7,10]. Thus, regulation of microglial activation may play an important role in enabling the parasite to (1) suppress host inflammation and immunity as well as the development of the asymptomatic phase and (2) contain damaging inflammation in the symptomatic phase of NCC.

Recognition of external stimuli by cells is mediated through a wide variety of pattern recognition receptors (PRRs). The Toll-like receptor (TLR) family of molecules is the major class of PRRs instrumental in the regulation of the host immune response [11–13]. Indeed, our findings using a murine experimental model of NCC suggest that the TLR signaling pathway plays a prominent role in the development of the initial hyperinflammatory response, which in turn contributes to the neuropathology and disease severity in NCC [14]. Important unanswered questions are (1) whether neurocysticercal antigens inhibit TLR signaling-induced inflammatory cytokine production in microglia and (2) what are the mechanisms involved in this process. Helminth-induced modulation of microglial activation and regulation of production of inflammatory mediators from these brain cells is poorly understood. In this regard, the first step in agonist-induced activation of downstream signaling pathways by the pathogen or pathogen-associated molecular patterns involves immediate release of Ca^2+^ from intracellular endoplasmic reticulum (ER) stores, followed by subsequent store-operated Ca^2+^ entry (SOCE) across the plasma membrane (PM) [15]. Such Ca^2+^ influx could in turn culminate in the induction of the host inflammatory response [16]. The present study is focused on determining the immunosuppressive effect of helminth secretory or soluble factors (HSFs) to regulate TLR ligand-induced activation of the Ca^2+^ signaling pathway in microglia. Our results indicate that HSFs downregulate the agonist-induced inflammatory response and the activation of SOCE channels (transient receptor potential channel 1 (TRPC1) and ORAI Ca^2+^ release-activated Ca^2+^ modulator 1 (ORAI1)) and associated signaling pathways in microglia. This process likely plays an important role in regulating the initiation of the inflammatory responses in the nervous tissue during pathogenic conditions involving helminth parasites.

## Experimental procedures

### Animals and antigens

Maintenance of the animals and tissue collection for performing experiments were conducted according to the guidelines of the University of North Dakota system Institutional Animal Care and Use Committee, the US Department of Agriculture and the National Institutes of Health. The female BALB/c or C57BL/6 mice used in this study were purchased from Charles River Laboratories (Wilmington, MA, USA). *Mesocestoides corti* (*M. corti*) was kindly provided by Dr. De’Broski R Herbert (Division of Immunobiology, Cincinnati Children’s Research Foundation, Cincinnati, OH, USA). *M. corti* metacestodes were maintained in the peritoneal cavity of BALB/c mice by serial intraperitoneal infection for propagation [14,17]. HSFS consisting of *M. corti* soluble factors was prepared from *M. corti* metacestodes in phosphate-buffered saline (PBS). Briefly, isolated metacestodes from the peritoneal cavity of infected mice were washed with 8 volumes of ice-cold Hanks’ Balanced Salt solution eight to ten times. *M. corti* metacestodes were suspended in 5 volumes of PBS, incubated on ice for 10 minutes and pelleted by centrifugation at 500 × *g* for 5 minutes, and then the supernatant was aspirated. The pelleted metacestodes were suspended in 5 volumes of PBS with protease inhibitors, and the above-mentioned procedure was repeated two more times, but additionally with freezing and thawing of the metacestodes. The supernatants were pooled together, passed through 0.2-μm filters for sterilization and used as HSFs.

### Primary microglia maturation and activation

Microglia were derived from postnatal day 1 (P1) C57BL/6 mouse brains as previously described [18,19]. Briefly, the cortex (free of meninges) was taken from P1 mice and then isolated and trypsinized. Cells were plated onto tissue culture plastic in Dulbecco’s modified Eagle’s medium/Nutrient Mixture F-12 with l-glutamine (DMEM/F-12; Invitrogen, Carlsbad, CA, USA) containing 10% heat-inactivated fetal bovine serum and 5% heat-inactivated horse serum, and half the medium changed every third day. After about 14 days, the cultures were shaken vigorously for 30 minutes at 120 rpm on a rotary shaker to remove microglia. Microglial purity was routinely determined to be approximately 90% cells by immunofluorescence microscopy using a specific marker (Iba1 or CD11b) and by negative staining for glial fibrillary acidic protein, a marker for astrocytes. To test the inhibitory effect of HSFs on the secretion of proinflammatory cytokines, the cells were plated at 8 × 10^4^ cells per well in 96-well flat-bottomed plates and were stimulated with medium alone or in the presence of HSFs, TLR ligands or HSF/TLR ligands. Culture supernatants were collected 24 hours after stimulation, and tumor necrosis factor α (TNF-α) and interleukin 6 (IL-6) were measured by enzyme-linked immunosorbent assay according to the manufacturer’s instructions (BD OptEIA; BD Biosciences, San Jose, CA, USA).

### Assessment of microglia by MTT assay

Primary microglia were treated with medium alone, HSF, lipopolysaccharide (LPS), or HSF and LPS, and cell viability was measured using a 3-(4,5-dimethyl-2-thiazolyl)-2,5-diphenyl-2H-tetrazolium bromide (MTT) assay according to the manufacturer’s instructions (Sigma-Aldrich, St Louis, MO, USA).

### Calcium measurements

Microglia were plated at 1 × 10^6^ cells on collagen- and poly-d-lysine–coated, 35-mm, glass-bottomed culture dishes (MatTek, Ashland, MA, USA) at 37°C. After a 2- to 4-hour incubation, cells were washed twice with standard extracellular solution (SES) (145 mM NaCl, 5 mM CsCl, 1 mM MgCl_2_, 1 mM CaCl2, 10 mM 4-(2-hydroxyethyl)piperazine-1-ethanesulfonic acid (HEPES), 10 mM glucose, pH 7.4, with NaOH, containing 0.02% soybean trypsin inhibitor and 0.1% bovine serum albumin and collagenase P (2.5 mg/8 ml of buffer)) for 15 to 20 minutes at 37°C. Cells were incubated in SES buffer containing 2 μM Fura-2 acetoxymethyl ester (Fura-2 AM) for 45 to 60 minutes at 37°C [20,21]. Before we performed Ca^2+^ measurements, the culture dishes were washed with and placed in Ca^2+^-free SES buffer. Cells were stimulated with medium alone or with 25 μg/ml HSF for 20 minutes before addition of thapsigargin (Tg) or LPS. Tg is a sarco(endo)plasmic reticulum Ca2+ ATPase pump blocker that does not engage any PRRs on the cell membrane. Fluorescence measurements were performed by imaging the Fura-2 AM–loaded microglia using an Olympus IX50 inverted microscope (Olympus America, Center Valley, PA, USA) with excitation light provided by a Polychrome IV monochromator (TILL Photonics, Hillsboro, OR, USA) [20,21]. Images were acquired using a Photometrics CoolSNAP HQ charge-coupled device camera (Photometrics, Tucson, AZ, USA) and MetaFluor software (Molecular Devices, Sunnyvale, CA, USA). Relative average intracellular Ca^2+^ concentration values are from at least 30 to 40 microglia and are representative of results obtained in at least three or four individual experiments.

### Electrophysiology

All electrophysiological experiments were performed according to a previously published protocol [20–22]. Coverslips with freshly isolated microglia were transferred to the recording chamber and perfused continually through a custom-designed, gravity-driven, speed-controlled system at a rate of 5 ml/min with an external Ringer’s solution (145 mM NaCl, 5 mM KCl, 1 mM MgCl_2_, 1 mM CaCl_2_, 10 mM HEPES, 10 mM glucose, pH 7.4, with NaOH). The patch pipette had resistances between 3 mΩ and 5 mΩ after filling with the standard intracellular solution (145 mM cesium methane sulfonate, 8 mM NaCl, 10 mM MgCl_2_, 10 mM HEPES, 10 mM ethylene glycol tetraacetic acid, pH 7.2, with CsOH). Osmolarity for all solutions was adjusted with d-mannitol to 305 ± 5 mmol/kg using a VAPRO pressure osmometer (Wescor/ELITech Group, Princeton, NJ, USA). Patch-clamp experiments were performed in the tight-seal, whole-cell configuration at room temperature (22°C to 25°C) using an Axopatch 200B amplifier (Molecular Devices). Voltage ramps ranging from −90 to 90 mV over a period of 1 second were imposed every 4 seconds from a holding potential of 0 mV and digitized at a rate of 1 kHz. A liquid junction potential of <8 mV was not corrected, and capacitive currents and series resistance were determined and minimized. For analysis, the first ramp was used for leak subtraction for the subsequent current records. Currents were normalized to the initial size of the cell to obtain current densities (pA/pF).

### Western blotting

Microglia at 1.5 × 10^6^ per well in 12-well plate were pulsed with medium alone or with HSF for 20 minutes, followed by addition of LPS or medium alone. Cells were solubilized in 100 μl of 2× SDS-PAGE sample buffer, and 30 μl of extracts were resolved on 12% SDS-PAGE, transferred onto an Immobilon polyvinylidene fluoride membrane (Bio-Rad Laboratories, Hercules, CA, USA) for immunostaining. Blots were incubated with primary antibodies (Abs) against phosphorylated nuclear factor κB (NF-κB; p65 subunit), extracellular signal-regulated kinase 1/2 (ERK1/2) and c-Jun N-terminal kinase (JNK) or acetylated NF-κB p65 (Cell Signaling Technology, Beverly, MA, USA). For loading controls, blots were incubated with primary Abs against a ubiquitously expressed protein, glyceraldehyde 3-phosphate dehydrogenase (GAPDH). Bands were visualized using corresponding secondary horseradish peroxidase–conjugated Abs and standard enhanced chemiluminescence (Amersham Biosciences, Piscataway, NJ, USA).

### Coimmunoprecipitation and Western blot analyses

Coimmunoprecipitation and Western blot analyses were carried out as described elsewhere [23,24]. Microglia were pulsed with medium alone or with HSF at 25 μg/ml for 20 minutes, followed by addition of 2 μM Tg, LPS or dimethyl sulfoxide (0.1% vol/vol) for 5 minutes at 37°C, and then they were washed with ice-cold PBS and lysed in 1× radioimmunoprecipitation assay buffer supplemented with 0.1% SDS, 1% Triton X-100, 20% glycerol, 1 mM phenylmethylsulfonyl fluoride and 1× protease and phosphatase inhibitors. Protein concentrations were adjusted to 1 mg/ml and immunoprecipitated with Abs against stromal interaction molecule 1 (anti-STIM1) [25]. Immunocomplexes were separated using Protein A Agarose Plus beads (Pierce Biotechnology, Rockford, IL, USA), eluted with 50 μl of 1× SDS dye and resolved in 4–12% SDS-PAGE gels (Bio-Rad Laboratories), followed by Western blotting as described previously using anti-TRPC1, anti-ORAI1 and anti-STIM1 Abs [23,24].

### Statistical analysis

We used the Student’s *t*-test and one-way analysis of variance for comparison of mean values in different groups (SigmaPlot 8.0 software (Systat Software, San Jose, CA, USA)). A *P*-value <0.05 was considered to be statistically significant.

## Results

### Helminth soluble factors inhibit Toll-like receptor ligand-induced activation of microglia

**Figure 1:**
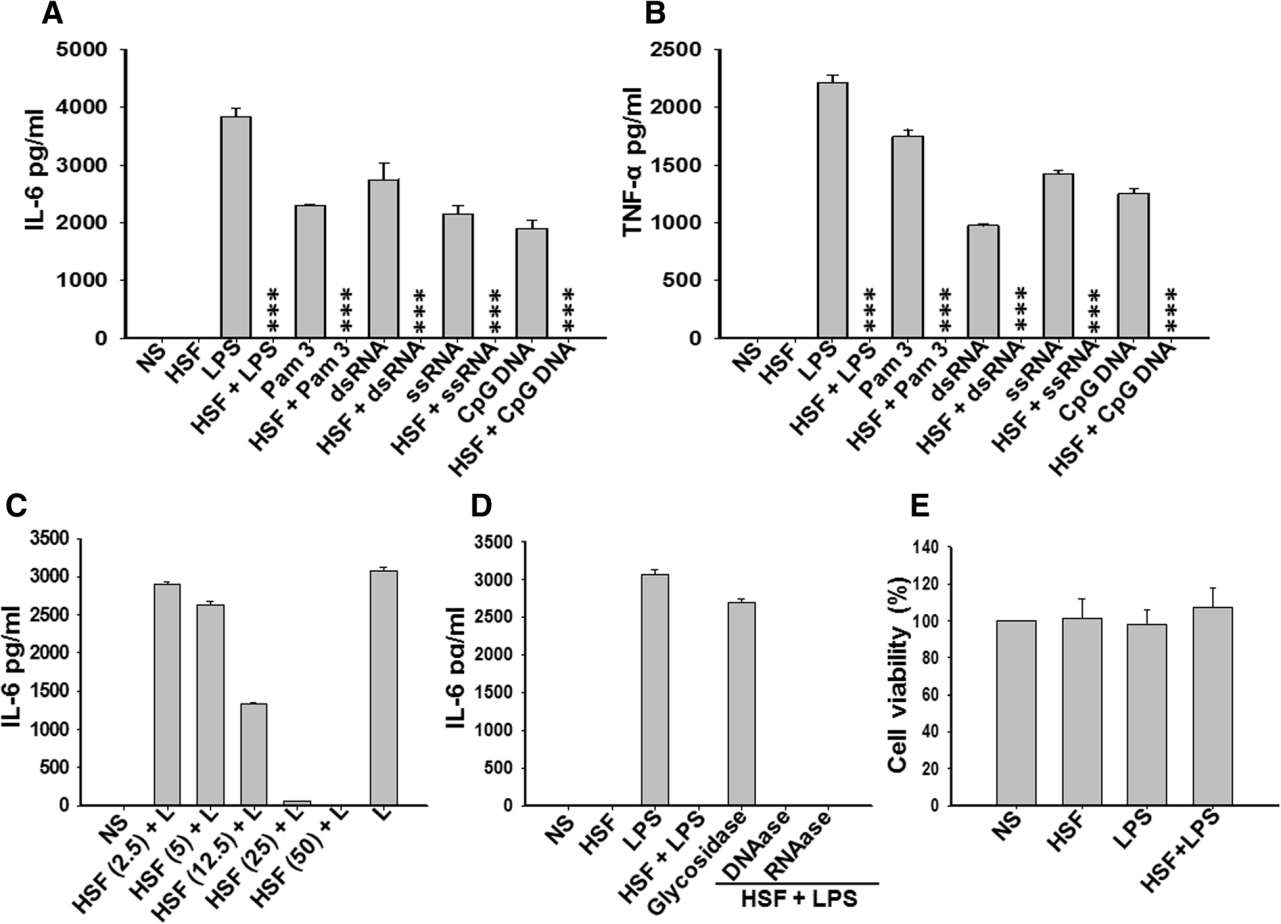
**Effect of helminth soluble factors on cytokine production by microglia.** Microglia were pulsed with medium alone, helminth soluble factors (HSFs) at 25 μg/ml or various Toll-like receptor (TLR) ligands (lipopolysaccharide (LPS; 10 ng/ml), Pam3Cys4 (Pam3; 10 ng/ml), double-stranded RNA (dsRNA; 10 ng/ml), single-stranded RNA (ssRNA; 10 ng/ml), CpG site DNA (1 μM) or HSFs) before the addition of the respective TLR agonists in the medium. Cells were cultured for a 24-hour period. The cytokine contents of interleukin 6 (IL-6) **(A)** and tumor necrosis factor α (TNF-α) **(B)** in culture supernatants were assayed by using specific sandwich enzyme-linked immunosorbent assays (ELISAs) as recommended by the manufacturer (BD Biosciences or R&D Systems (Minneapolis, MN, USA)). The mean ± SE concentration of cytokines in five independent experiments for IL-6 and three independent experiments for TNF-α was determined. **(C)** Microglia were pulsed with medium alone or with HSF at various concentrations (2.5 μg/ml, 5 μg/ml, 12.5 μg/ml, 25 μg/ml or 50 μg/ml) before the addition of LPS (L; 10 ng/ml) to the medium. The cytokine contents of IL-6 in culture supernatants were assayed at 24 hours as described above. **(D)** Cells were pulsed with HSF with or without RNase, DNase or glycosidase (O-glycosidase; Sigma-Aldrich) to modify glycan moieties 20 minutes prior to addition of LPS at 10 ng/ml. Culture supernatants were collected after 24 hours, and IL-6 concentration was measured by ELISA. **(E)** Microglia were pulsed with medium alone, LPS (10 ng/ml) and HSF (25 μg/ml) with or without LPS (10 ng/ml) in the medium for 24 hours. An MTT assay was performed to detect the cell viability. Absorption values at 570 nm were normalized to untreated cells. Data are presented as the percentage of untreated cells, with viability referred to as 100%. Significant differences were measured by Student’s *t*-test and are denoted by asterisks (***P < 0.001). NS, Nonstimulated.

The effect of HSFs on activation of primary microglia was tested *in vitro*. HSFs alone did not modulate expression of the inflammatory cytokines IL-6 and TNF-α (Figure 1 and data not shown). We then tested their immunosuppressive effects on agonist-induced activation of microglia. As expected, exposure of microglia to the TLR ligands Pam3Cys4 (TLR1/2 ligand), double-stranded RNA (dsRNA; TLR3 ligand), LPS (TLR4 ligand), single-stranded RNA (ssRNA; TLR7/8 ligand) and CpG site DNA (TLR9 ligand) led to upregulated secretion of the inflammatory cytokines IL-6 and TNF-α (Figure 1A and 1B). This was correlated with complete inhibition of TLR activation-induced mRNA expression by HSFs in microglia cells (data not shown). Interestingly, coexposure with HSFs led to complete inhibition of TLR ligand-induced secretion of IL-6 and TNF-α cytokines by microglia (Figure 1A and 1B). HSFs inhibited LPS-induced cytokine secretion in a concentration-dependent manner (Figure 1C). This inhibition by HSFs was abolished when glycosidase was used to modify the glycan constituent (Figure 1D), indicating the specificity of this effect and possible involvement of glycan moieties in this process. To further exclude nonspecific effects of HSFs on cell death, which may indirectly contribute to HSF-induced inhibition of agonist-elicited activation, the cell viability of microglia was assessed by MTT assay. A similar level of cell viability was observed after stimulation of microglia with HSFs alone or upon exposure to LPS in the presence or absence of HSFs (Figure 1E). This further attested to the specificity of the immunosuppressive effect of HSFs. These results demonstrate that, though HSF alone does not elicit microglial activation, it efficiently inhibits TLR ligand-induced production of inflammatory mediators in microglia.

### HSF inhibits activation of LPS-induced NF-κB and MAPK activity

**Figure 2:**
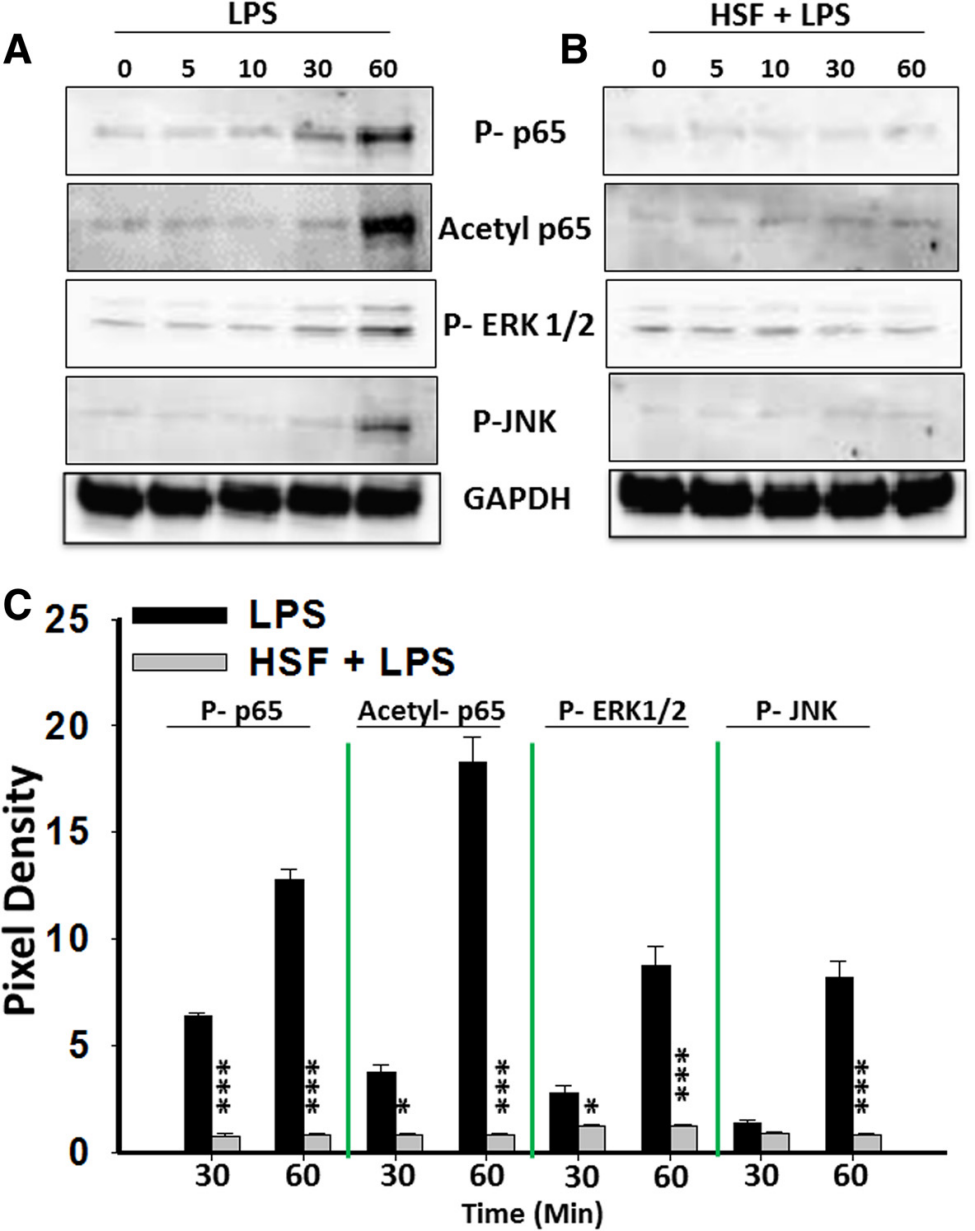
**Helminth soluble factor–mediated modulation in lipopolysaccharide-induced signaling in microglia.** Microglia were pulsed with medium alone, with lipopolysaccharide (LPS) **(A)** or with helminth soluble factor (HSF) and LPS **(B)**. Equal amounts of lysates from microglia pulsed for the periods of time shown were electrophoretically separated, blotted and probed with antibodies specific for phosphorylated nuclear factor κB (NF-κB) p65 subunit (P-p65), p38, c-Jun N-terminal kinase (P-JNK) and extracellular signal-regulated kinase 1/2 (P-ERK1/2) or acetylated NF-κB p65, whereas antibody against glyceraldehyde 3-phosphate dehydrogenase (GAPDH) was used as a loading control. The data shown are representative of three independent experiments. **(C)** The average pixel intensity of the respective bands at 30 minutes and 60 minutes from three independent experiments was measured. This was done using Quantity One 1-D version 4.6.7 software (Bio-Rad Laboratories). Significant differences were measured by using Student’s *t*-test, and significant values are denoted by asterisks (*P < 0.05 and ***P < 0.001).

The cascade of events that follows the TLR activation that leads to NF-κB and mitogen-activated protein kinase (MAPK) activation culminates in the production of inflammatory mediators [26,27]. We examined the effect of HSF on LPS-induced NF-κB and MAPK activity by measuring phosphorylation and acetylation of NF-κB p65, as well as phosphorylation of JNK and ERK1/2, all of which are important downstream signaling components activated by TLR ligation [27]. LPS stimulation increased both the phosphorylation and acetylation of p65 (Figure 2A and 2C), which was inhibited by HSF (Figure 2B and 2C). Similarly, LPS stimulation increased the phosphorylation of both JNK and ERK1/2 (Figure 2A and 2B), which was decreased by HSF exposure (Figure 2B and 2C). Further, the GAPDH level was unaffected in control cells or in those stimulated with LPS, HSF or LPS and HSF together (Figure 2A and 2B). Thus, HSF-induced suppression of the LPS-initiated inflammatory response correlated with an inhibition of induced NF-κB and MAPK activity.

### Helminth soluble factor inhibits activation induced Ca^2+^ entry

**Figure 3:**
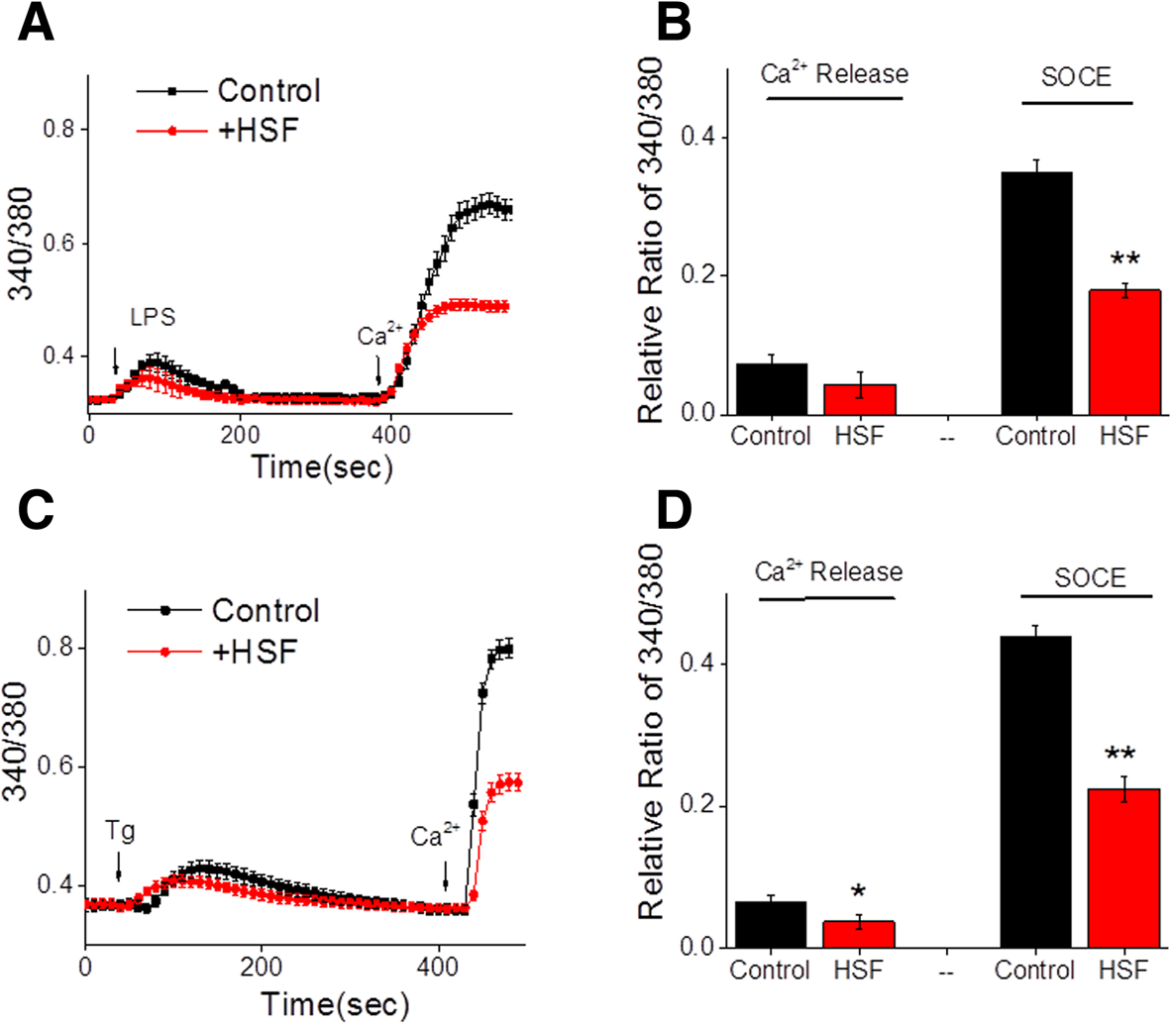
**Helminth soluble factor inhibits agonist induced Ca** ^**2+**^ **release and Ca** ^**2+**^ **entry.** Fura-2 acetoxymethyl ester fluorescence measurements in microglia. **(A)** Cells were pulsed with medium alone, lipopolysaccharide (LPS) at 10 ng/ml (Control) or pretreated helminth soluble factor (HSF) at 25 μg/ml for 20 minutes before the addition of LPS to the medium (+HSF). Average analog plots of the fluorescence ratio (340/380 nm) from an average of 40 to 50 cells are shown. **(B)** The bar graph indicates the average data for Ca^2+^ release (left peak) and store-operated Ca^2+^ entry (SOCE) (right peak) under these conditions in microglia pulsed with LPS or HSF + LPS. Asterisks indicate significance (**P ≤ 0.01). **(C)** Cells were pulsed with medium alone, thapsigargin (Tg) at 1 μM or pretreated HSF at 25 μg/ml for 20 minutes before the addition of Tg at 1 μM. Analog plots of the fluorescence ratio (340/380 nm) from an average of 30 to 40 cells are shown. **(D)** The bar graph indicates the average data on Ca^2+^ release (left peak) and Ca^2+^ entry (right peak) under these conditions in microglia pulsed with Tg or HSF + Tg. Asterisks indicate significance (*P ≤ 0.05, ** *P* ≤ 0.01).

Ca^2+^ entry through SOCE channels has been implicated in the activation of signaling pathways essential for the inflammatory response elicited after cellular exposure to stimuli [28,29]. Thus, we performed Ca^2+^ imaging and electrophysiological experiments to determine whether HSFs affect the Ca^2+^ signaling pathway in primary microglia. Exposure to HSFs alone did not produce any substantial changes in cytosolic Ca^2+^ levels in microglia (data not shown). However, LPS stimulation of microglia in a Ca^2+^-free buffer resulted in an increase in cytosolic Ca^2+^ levels due to the release of internal ER Ca^2+^ (left peak (arrow) in Figure 3A; quantitative data are shown in Figure 3B) and in terms of Ca^2+^ influx through the PM (right peak (arrow) in Figure 3A; quantitative data are shown in Figure 3B) due to ER store depletion-induced SOCE. HSF-treated microglia showed a significant decrease in the cytosolic Ca^2+^ level in response to LPS stimulation (Figure 3). This was evidenced by a reduced LPS-stimulated cytosolic Ca^2+^ influx without a significant change in the ER Ca^2+^ levels (Figure 3), suggesting that HSFs block activation-induced Ca^2+^ channel activation in the PM and associated Ca^2+^ influx. Next, to test the specificity of HSFs affecting induced Ca^2+^ influx and/or channel activation, we examined if HSFs have any effect on Tg-induced activation of intracellular Ca^2+^ turnover. Stimulation of microglia with Tg induced both Ca^2+^ release at the ER and Ca^2+^ influx at the PM (left peak (arrow) in Figure 3C). Interestingly, exposure of microglia to HSFs abolished this Tg-induced Ca^2+^ turnover (right peak (arrow) in Figure 3C). This inhibition of Tg-induced SOCE through the PM by treatment with HSFs was statistically significant (Figure 3D).

**Figure 4:**
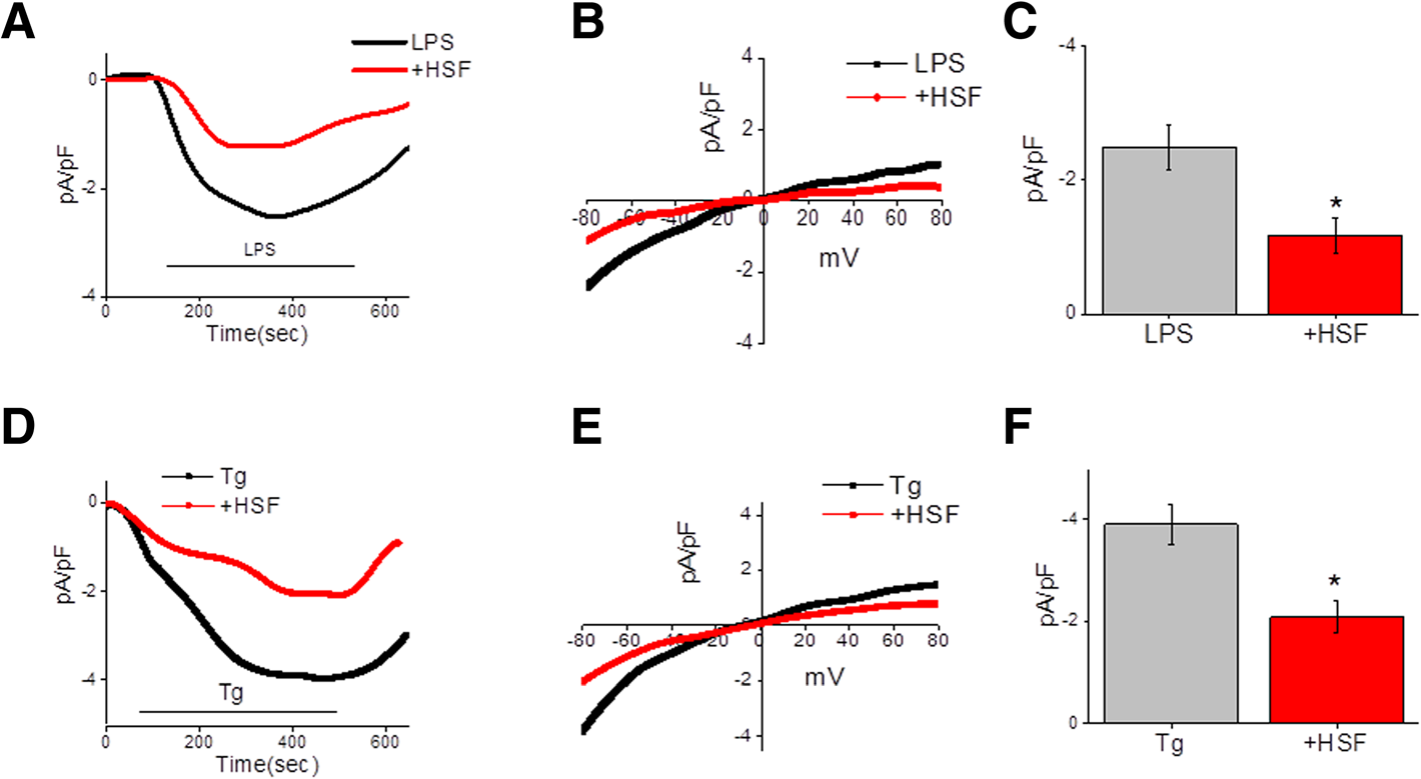
**Helminth soluble factors inhibit agonist-induced TRPC1/ORAI1 store-operated Ca** ^**2+**^ **entry.** Microglial cells were used for electrophysiological recordings, and both lipopolysaccharide (LPS)- and thapsigargin (Tg)-induced currents were evaluated in control cells and cells treated with helminth soluble factor (HSF) (25 μg/ml for 20 minutes). **(A)** Inward currents were induced upon addition of LPS (10 ng/ml). The holding potential for current recordings was −80 mV in both control and HSF-treated cells. **(B)** Respective current-voltage (I-V) curves using the ramp protocol in control and HSF-treated cells stimulated with LPS are shown. **(C)** Average of eight to ten recordings with current intensity at −80 mV are shown. **(D)** Inward currents induced by the addition of Tg (1 μM) in control cells and cells pretreated with HSF (25 μg/ml for 20 minutes). **(E)** The respective I-V curves under these conditions are shown. **(F)** Average of eight to ten recordings of current intensity at −80 mV are shown. *P ≤ 0.05.

**Figure 5:**
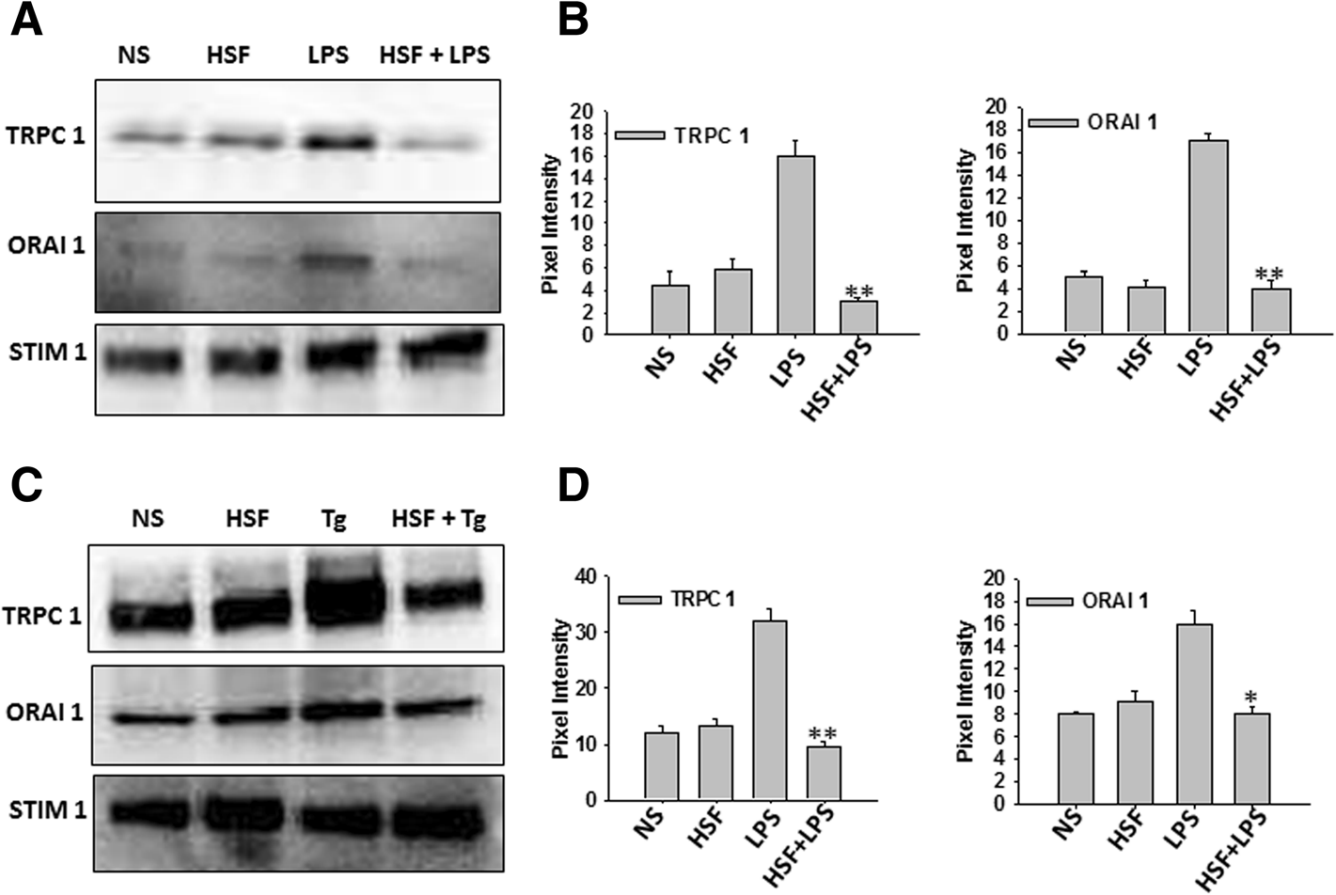
**Helminth factors impair store-mediated TRPC1-STIM1 and ORAI1-STIM1 channel assembly. (A)** Immunoprecipitation using anti-STIM1 antibodies, of equal amount of lysates from control nonstimulated (NS) cells, treated with or without lipopolysaccharide (LPS), or cells treated with helminth soluble factors (HSFs) (25 μg/ml for 20 minutes) and stimulated with or without LPS. Immunoblotting was performed using anti-TRPC1, anti-ORAI1 and anti-STIM1 antibodies at 1:1,000 dilution each. ORAI1, ORAI Ca^2+^ release-activated Ca^2+^ modulator 1; STIM1, Stromal interaction molecule 1; TRPC1, Transient receptor potential channel 1. **(B)** Bar graphs show the densitometric values of TRPC1 and ORAI1. Values are expressed as mean ± SE. **P < 0.05 versus HSF–untreated, LPS-stimulated cells. **(C)** Immunoprecipitation using anti-STIM1 antibodies in amounts equal to lysates from control NS cells, treated with or without thapsigargin (Tg), or cells treated with HSF (25 μg/ml for 20 minutes) and stimulated with or without Tg and immunoblotted using the respective antibodies. **(D)** The average pixel intensity of the respective protein bands from three independent experiments was measured using Quantity One 1-D version 4.6.7 software (Bio-Rad Laboratories). Asterisks indicate significance (*P ≤ 0.05, **P ≤ 0.01).

Next, electrophysiological recordings were performed to identify the SOCE channel involved in microglial cells. As shown in Figure 4A and 4B, addition of LPS initiated a nonselective Ca^2+^ current that reversed between 0 to 5 mV and was partially inward-rectifying. Furthermore, the Ca^2+^ current was inhibited in cells that were pretreated with the HSFs (Figure 4A and 4B), a finding which was statistically significant (Figure 4C). Additionally, the current-voltage (I-V) properties of the channel were different, as observed for both TRPC1-dependent store-operated current (*I*_SOC_) and ORAI1-mediated Ca^2+^ release-activated Ca^2+^ current [25], suggesting that the channel is probably a mixed channel that consists of TRPC1 and ORAI1. Exposure of cells to HSF not only decreased Ca^2+^ currents (Figure 4A, 4B and 4C) but also made the current slightly inward-rectifying (Figure 4B), again suggesting that this might be a mixed channel. Furthermore, addition of Tg, which also initiates ER Ca^2+^ release and induces SOCE, was significantly decreased in microglial cells exposed to HSF (Figure 4D, 4E and 4F). Furthermore, similarly to LPS, the I-V properties of the channel-activated/Ca^2+^ currents induced by Tg and inhibited by HSF in microglia are consistent with both TRPC1- and ORAI1-dependent Ca^2+^ current. To confirm the inhibition of TRPC1 and ORAI1 functions in the Ca^2+^ influx by HSF, biochemical analyses were performed. In this regard, interaction of STIM1 (the ER Ca^2+^ sensor protein) with channels such as TRPC1 and ORAI1 on PM is essential for their activation and subsequent SOCE [22]. Thus, coimmunoprecipitation was performed using STIM1 Abs to pull down PM proteins bound to it in microglia stimulated with LPS or Tg with or without HSF. This was followed by immunoblot analysis in which we specific Abs against TRPC1 and ORAI1. As shown in Figure 5, the unstimulated cells and the cells exposed to HSF alone did not display any difference in TRPC1-STIM1 or ORAI1-STIM1 association. On the other hand, microglial stimulation with LPS (Figure 5A and 5B) or Tg (Figure 5C and 5D) alone led to a significant increase in TRPC1-STIM1 or ORAI1-STIM1 association. However, upon treatment with HSF, both LPS- and Tg-induced TRPC1-STIM1 or ORAI1-STIM1 association reverted back to basal control levels (Figure 5). Further, immunoblot analysis revealed that the stimulation had no measurable effect on STIM1 levels, as compared to the untreated microglia (Figure 5A and 5C). Together, these data confirm that HSF inhibits agonist-induced increases in TRPC1- and ORAI1-dependent Ca^2+^ influx through PM in microglia.

## Discussion

Inflammatory disorders can be triggered by overactive immune responses directed against host and/or self-tissues. It is now evident that a wide range of neuroinflammation-associated disorders, such as infections of the CNS, stroke and cognitive disorders, and even classic mental illnesses, such as schizophrenia and depression, can be triggered and/or exacerbated by overactive immune responses against nervous tissues [30–32]. Accordingly, agents that suppress inflammatory responses may provide a means by which to ameliorate the pathologies associated with many of these disorders. In this regard, it is remarkable that parasitic helminths are master regulators of host inflammatory response and pathology [33]. In the case of NCC, it is presumed that factors associated with the live stage of helminths likely inhibit CNS inflammation and hence induce an asymptomatic phase lasting 3 to 5 years [1,26]. In contrast, the loss of immunosuppressive effects when the parasite dies is thought to lead to an uncontrolled, detrimental, hyperinflammatory response that contributes to tissue pathology and clinical manifestations such as epileptic seizures, strokes and severe neurological symptoms in NCC [2,6,14,26]. However, the molecular mechanisms by which the helminths and their products inhibit CNS inflammation are not well understood. In this study, we investigated the mechanism of helminth-induced inhibition of innate immune pathway activation in microglia. To our knowledge, this study is the first to demonstrate helminth-mediated inhibition of activation of ORAI1 and TRPC1 Ca^2+^ channel function in microglia. The results reported here strongly support the hypothesis that the downregulation of Ca^2+^ channel activation and associated signaling by helminths is involved in blocking innate immune pathway activation-induced inflammatory response in microglia and in the CNS in general.

The CNS is regarded as an immune-privileged site because of the absence of a defined lymphatic drainage system and the presence of the blood–brain barrier (BBB) [34]. However, the CNS possesses active immune processes and regulatory mechanisms. In this context, among all the nervous tissue cell types, microglia are thought to be the main cell type of the innate immune system in the brain and are commonly associated with immune reactions. During infection, recognition of microbial molecules by microglial PRRs leads to production of inflammatory mediators such as chemokines and cytokines [35,36]. Thus, activation of microglia plays an important role in leukocyte trafficking into the brain through its effects on the BBB, eventuating in pathogen-specific adaptive immune responses in the CNS [37]. However, aberrant regulation of microglia-elicited inflammatory responses has been implicated in the majority of neurodegenerative disorders [38–42]. In this regard, increasing evidence also indicates that, among the PRRs, the TLR family of molecules plays a major role in several inflammatory CNS pathologies [26–30]. TLR signaling leads to production of host inflammatory mediators, which in turn plays a significant role in leukocyte trafficking into the CNS [43]. Indeed, induction of TLRs in the infected and/or diseased brain for extended periods is integral to persistent inflammation and its associated pathology [37,44,45]. The results of our previous studies in murine models of NCC suggest that TLRs 1 through 13 exhibit differential expression and regulation in normal and NCC mouse brains, with TLRs 2, 3, 4, 6, 7, 8 and 9 expressed on CD11b + myeloid cells [46,47]. Moreover, MyD88-deficient mice display a reduction in infiltration by immune cells as well as proinflammatory cytokine responses in the CNS that coincides with reduced neuropathology and disease severity [14].

To the best of our knowledge, we are the first to report clear results showing that helminth factors directly inhibit both MyD88-dependent (exposure to LPS, Pam3Cys4, ssRNA or CpG site DNA) and MyD88-independent (exposure to dsRNA) TLR signaling pathway activation-associated inflammatory cytokine production in microglia. This inhibitory effect is dependent on the concentration of the immunosuppressive helminth factors and TLR ligands used. Interestingly, the inhibition of the LPS-induced effect by HSF was abrogated when was exposed to glycosidase, suggesting the specificity of the effect as well as the possible involvement of glycan factors in the parasite-induced immune modulation. Helminths (worms), in their pursuit to establish long-term infections and survival in the host, use glycans, which are abundant on their surface (tegument) and in their excretory and/or secretory products [47,48], to regulate and suppress host immune responses. Indeed, both *M. corti* and the human parasite *Taenia solium* are complex organisms, and, during the infection process, they release many molecules, including glycans [49–53]. Interestingly, in the human brain, the presence of glycan antigens in CD68+ myeloid cells (presumably macrophages and/or microglia) has been shown to correlate with a lack of surface expression of the activation and/or maturation marker major histocompatibility complex (MHC) class II [17,49]. During both human and murine NCC, the glycan antigens are released from the parasite and are taken up by immune cells (in areas around the metacestode) that typically display a lack of maturation of antigen-presenting cells by way of reduced expression of MHC class II [49]. We expect that novel immunosuppressive molecules in HSF (for example, glycans) are released from live parasites and have profound effects on the net proinflammatory response in NCC [1,54,55]. In contrast, our unpublished observations indicate that somatic antigen preparations from *M. corti* can induce proinflammatory cytokine production. This is supported by earlier studies showing that DNA and lipid antigens of parasites such as *Trypanosoma cruzi* and *Plasmodium falciparum* induce proinflammatory cytokine production [56,57]. In NCC patients, *T. solium* DNA could be detected in the cerebrospinal fluid of symptomatic NCC patients [58,59].

Characterization of the specific neurocysticercal antigens and host PRR interactions, as well as subsequent development of both inflammatory and immunosuppressive mechanisms, is a major focus of the current research in our laboratory. Taking our findings *in toto*, we speculate that the HSF-associated immunosuppressive helminth molecules are released from live larvae in the brain which interact with receptors on innate cells such as microglia and activate inhibitory signaling events, leading to failure of induction of the host inflammatory response. As the infection progresses, some of the parasites die, and the somatic antigens thus released activate a TLR-dependent inflammatory response. It is likely that the intensity of the polarized response elicited by these factors from live/dead parasites shapes the net inflammatory response in the CNS and determines the overall neuropathology and disease symptoms, or the lack thereof.

Cellular activation invariably involves Ca^2+^ signaling to regulate functions such as proliferation, migration, phagocytosis and gene transcription of inflammatory mediators [60]. Ca^2+^ entry across the PM is mediated by various ion channels, including SOCE and voltage-gated Ca^2+^ channels [61]. However, Ca^2+^ entry through SOCE channels seems to play an important role in activation of nonexcitable cells such as microglia [62,63]. We and others have previously demonstrated that the ORAI1–STIM1 and TRPC1–STIM1 interactions on PM mediate opening of the ORAI1 and TRPC1 channels and Ca^2+^ influx [25,64–70]. In our present study, we demonstrate that, though helminth factors themselves do not modulate basal levels of cytosolic Ca^2+^ turnover or channel activation, they can abolish TLR ligand (LPS)- and Tg-induced increases in Ca^2+^ influx as well as TRPC1–STIM1 and ORAI1–STIM1 interactions on PM of microglia. To the best of our knowledge, this report is the first to describe the involvement of TRPC1 SOCE channel activation in Ca^2+^ turnover in activated microglia. Moreover, the regulation of SOCE Ca^2+^ channel activation in microglia by parasites is likely a novel immunosuppressive mechanism that blocks the initiation of the inflammatory pathway in the CNS.

There is convincing evidence that Ca^2+^ entry through SOCE channels differentially activates different downstream signaling pathways. This is supported by our earlier studies showing contributions of TRPC1-mediated SOCE induced by TRPC1-STIM1 interactions to NF-κB activation [24,25]. The data we report here demonstrate that HSF-stimulated microglia exhibited significantly reduced levels of LPS-induced phosphorylation and acetylation of NF-κB (p65 subunit), as well as phosphorylation of ERK1/2 and JNK, as determined by immunoblot analysis. HSF efficiently inhibits both MyD88-dependent and MyD88-independent production of inflammatory mediators after TLR ligation. As a large body of literature indicates that TLR ligands activate protein kinase C (PKC) as well as other Ca^2+^-sensitive signaling mediators upstream of MAPK and NF-κB activation [71–81], one possibility is that HSF-induced inhibition of TRPC1 or ORAI1 SOCE may directly mediate downregulation of MAPK and NF-κB activation elicited by TLR ligands in both a MyD88-dependent and MyD88-independent manner, as a result of an upstream inhibition of the kinases, such as by the PKC that activates them. An additional possibility could be that downregulation of MAPK activities indirectly inhibit SOCE channel function. This hypothesis is supported by findings that the phosphorylation of STIM1 by ERK1/2 at residues Ser575, Ser608 and Ser621 was required to trigger full ORAI1 SOCE [82]. Along the same line, our results in the present study suggest that HSF-mediated inhibition of ERK1/2 activation and STIM1 phosphorylation may further augment the inhibitory effect on Ca^2+^ entry, particularly through the ORAI1 channel. Another interesting possibility could be that HSF directly binds to SOCE channels and inhibits their function, as our results from electrophysiological analysis show a HSF-induced instant decrease in *I*_SOC_-like Ca^2+^ current associated with TRPC1 SOCE channels in LPS- or Tg-stimulated cells (data not shown). However, further studies are required to characterize specific immunosuppressive parasitic molecules that might directly interact with SOCE channels to inhibit agonist-induced activation and Ca^2+^ entry. This is a major focus of the current research in our laboratory.

Collectively, the results of the present study demonstrate that HSFs suppress TLR ligand-induced MAPK and NF-κB activation, as well as inflammatory cytokine production, in primary microglia, which might have implications for the manifestation of the asymptomatic phase in NCC. Importantly, the negative regulation of TRPC1 SOCE and ORAI1 SOCE channel activation by HSFs in microglia represents a novel immunosuppressive mechanism to block the initiation of the inflammatory pathway in the CNS and is a completely unexplored area of research. Further characterization of immunosuppressive mechanisms involved could lead to novel therapeutic targets, as Ca^2+^-mediated, deregulated inflammatory response underlies the pathophysiological processes of many chronic inflammatory and autoimmune diseases.

## Abbreviations

Ab: Antibody
CNS: Central nervous system
ER: Endoplasmic reticulum
ERK1/2: Extracellular signal-regulated kinase 1/2
Fura-2AM: Fura-2 acetoxymethyl ester
GAPDH: glyceraldehyde 3-phosphate dehydrogenase
HSF: Helminth secretory or soluble factor
IL: Interleukin
JNK: c-Jun N-terminal kinase
LPS: Lipopolysaccharide
MAPK: Mitogen-activated protein kinase
MTT: 3-(4,5-dimethyl-2-thiazolyl)-2,5-diphenyl-2H-tetrazolium bromide
NCC: Neurocysticercosis
NF-κB: Nuclear factor κB
ORAI1: ORAI Ca^2+^ release-activated Ca^2+^ modulator 1
PBS: Phosphate-buffered saline
SOCE: Store-operated Ca^2+^ entry
STIM1: Stromal interaction molecule 1
Tg: Thapsigargin
TLR: Toll-like receptor
TNF-α: Tumor necrosis factor α
TRPC1: Transient receptor potential channel 1
